# Supplementary material for: Springtime winds drive Ross Sea ice variability and change in the following autumn
Source: Nat Commun. 2017 Sep 28;8:731. doi: 10.1038/s41467-017-00820-0 (PMC5620085; doi:10.1038/s41467-017-00820-0)
Supplement: Supplementary file 1 — Supplementary Information [file 41467_2017_820_MOESM1_ESM.pdf]

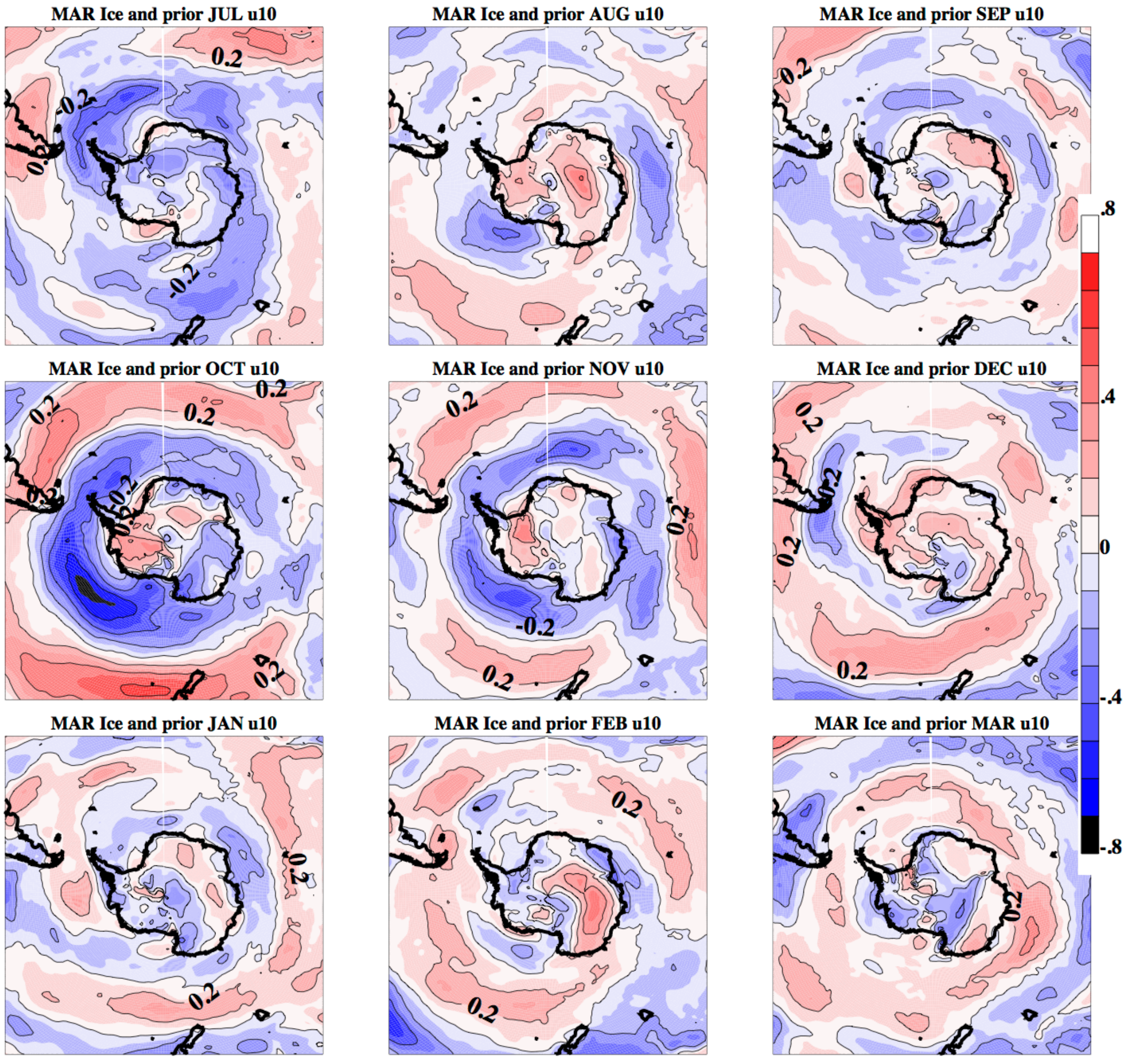

Supplementary Figure 1. Relationship of zonal winds and sea ice. Correlation of the 1979-2015 western Ross Sea March ice area with ERA-Interim 10m zonal winds for the previous nine months (from July in upper left panel to March in lower right panel). The lined contour interval is 0.2.

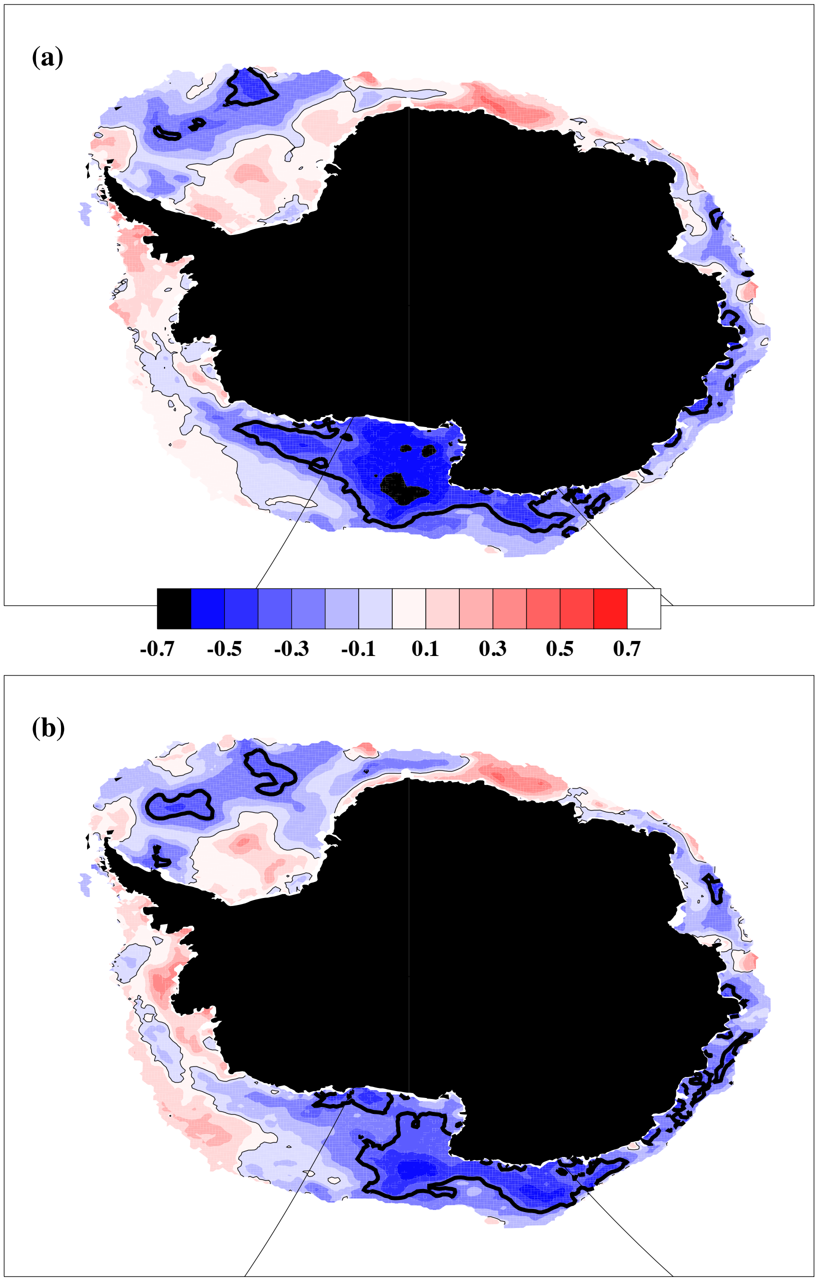

Supplementary Figure 2. Relationship of gridded sea ice with averaged zonal winds. The correlation of detrended gridded March sea ice concentration with detrended October zonal winds averaged over (a) the region of high correlation shown on Fig. 1d and (b) the region of high wind trends (black region on Fig. 5b). The thick black contour indicates the 95% significance level. The thin black contour indicates a correlation of zero. 150E and 200E, which are used to define the western Ross Sea region, are shown in thin black lines.

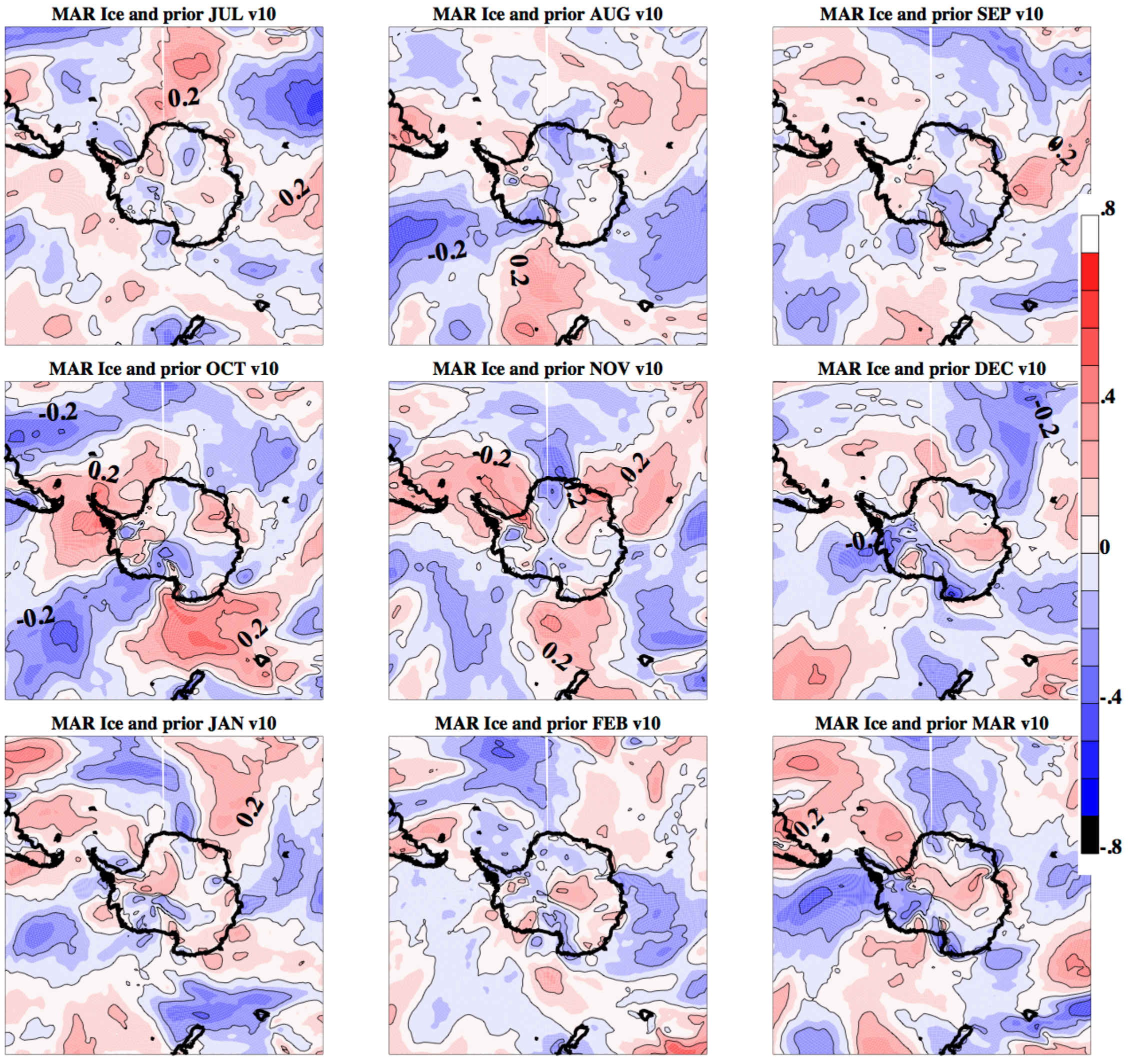

Supplementary Figure 3. Relationship of meridional winds and sea ice. Correlation of the 1979-2015 western Ross Sea March ice area with ERA-Interim 10m meridional winds for the previous nine months (from July in upper left panel to March in lower right panel). The lined contour interval is 0.2.

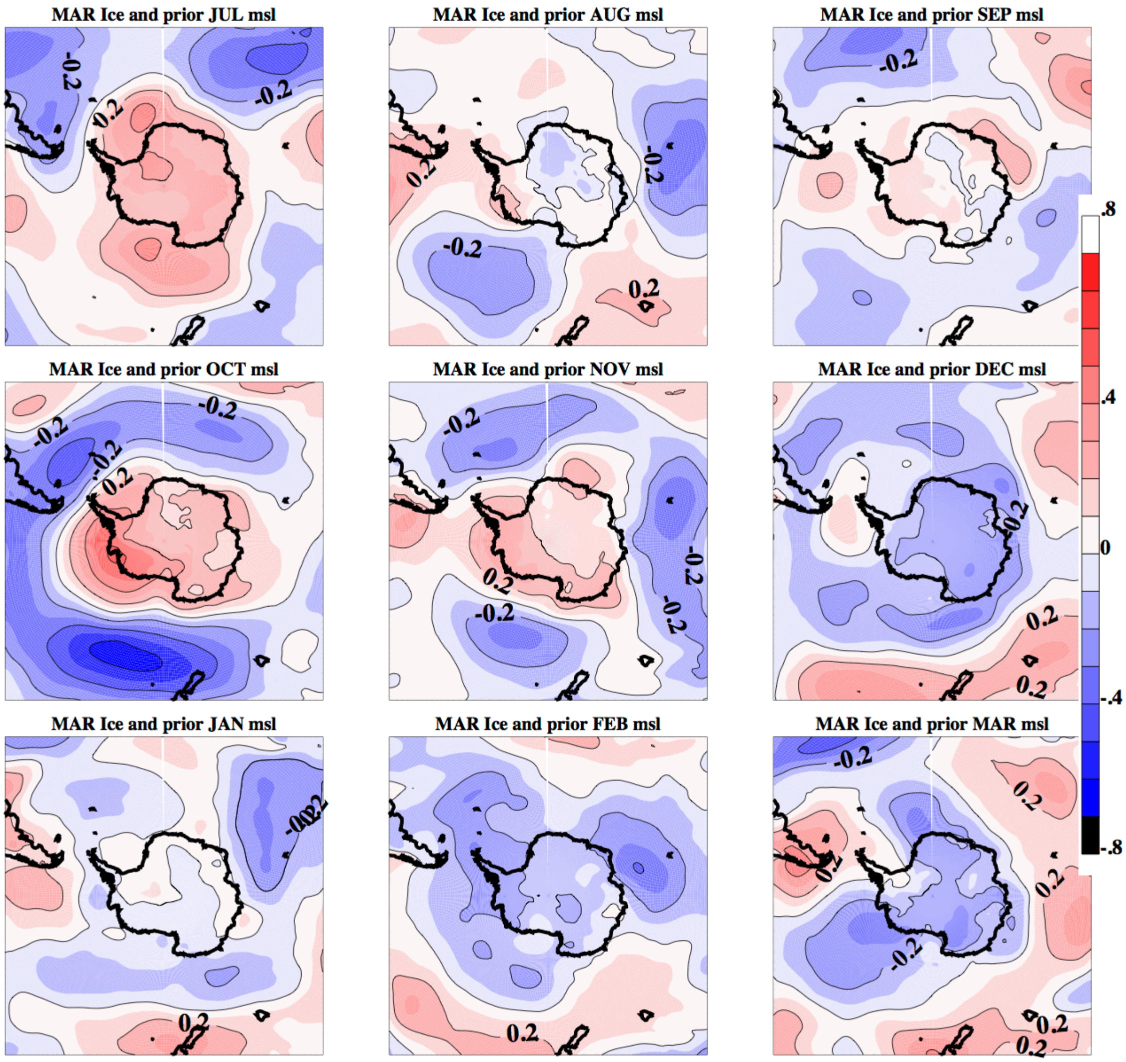

Supplementary Figure 4. Relationship of sea level pressure and sea ice. Correlation of the 1979-2015 western Ross Sea March ice area with ERA-Interim sea level pressure (SLP) for the previous nine months (from July in upper left panel to March in lower right panel). The lined contour interval is 0.2.

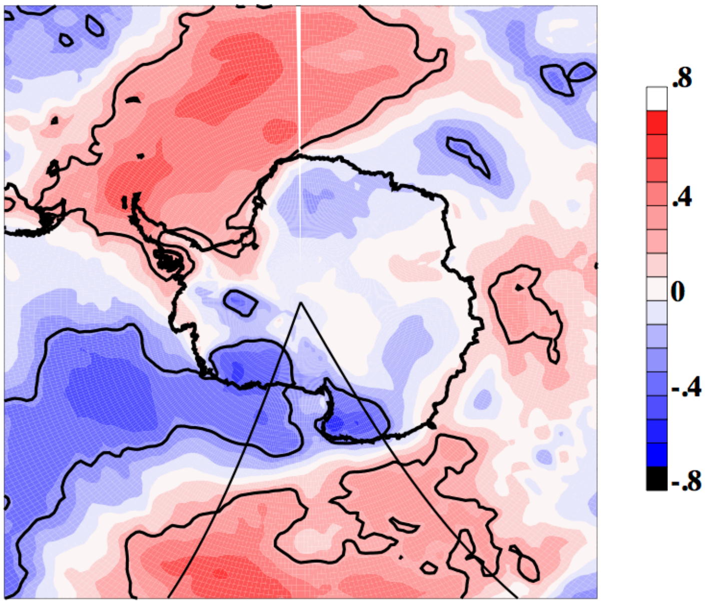

Supplementary Figure 5. Relationship of air temperature and zonal winds. The correlation of 1979-2015 gridded ERA-Interim October 2m air temperature and the October zonal wind index. The contour indicates values significant at the 95% level. The 150-200E region used for the sea ice analysis is indicated.

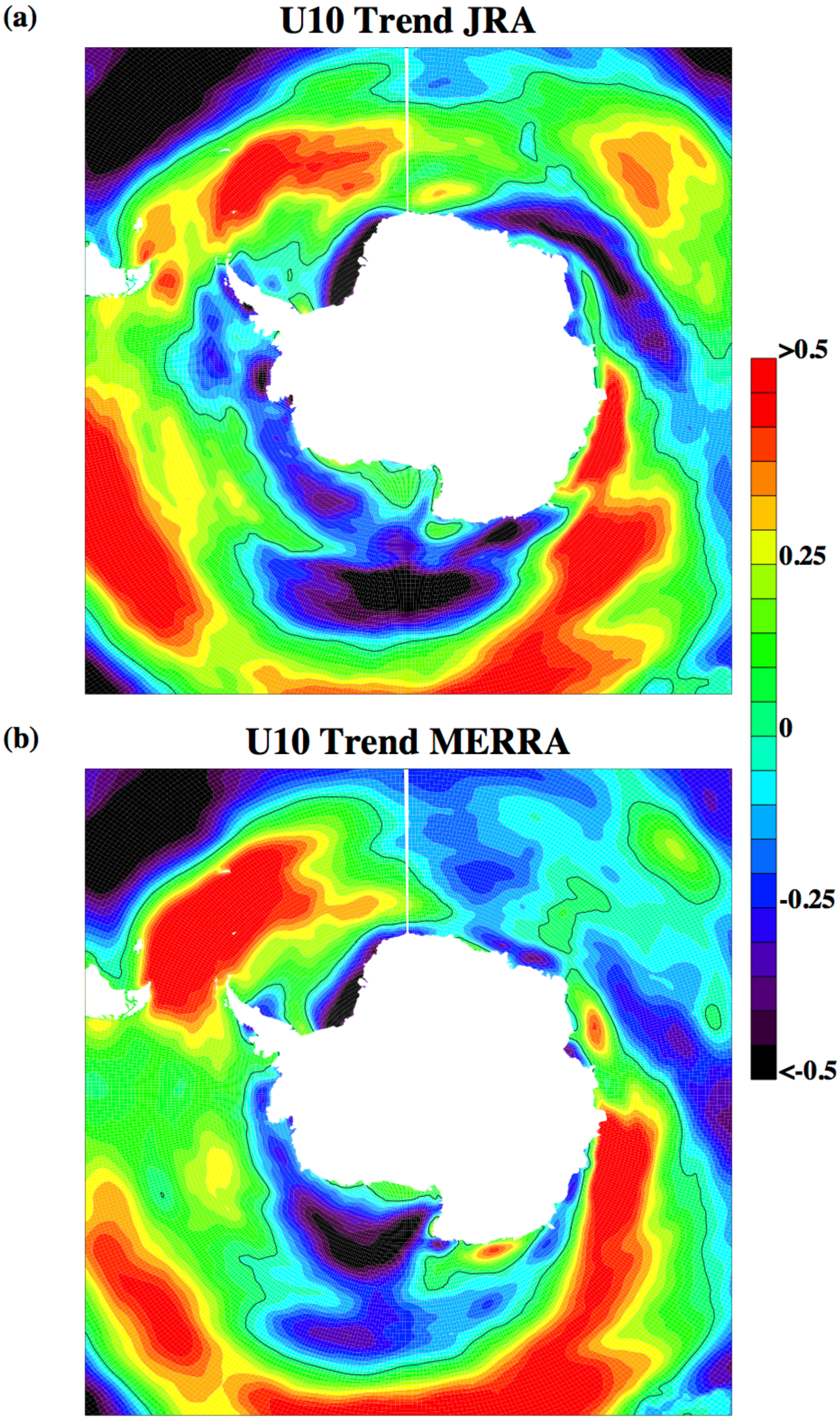

Supplementary Fig. 6. Zonal wind trends. Trends in the October zonal 10m winds in units of m/s per decade from (a) JRA-55 for 1979-2014 and (b) MERRA for 1979-2015.

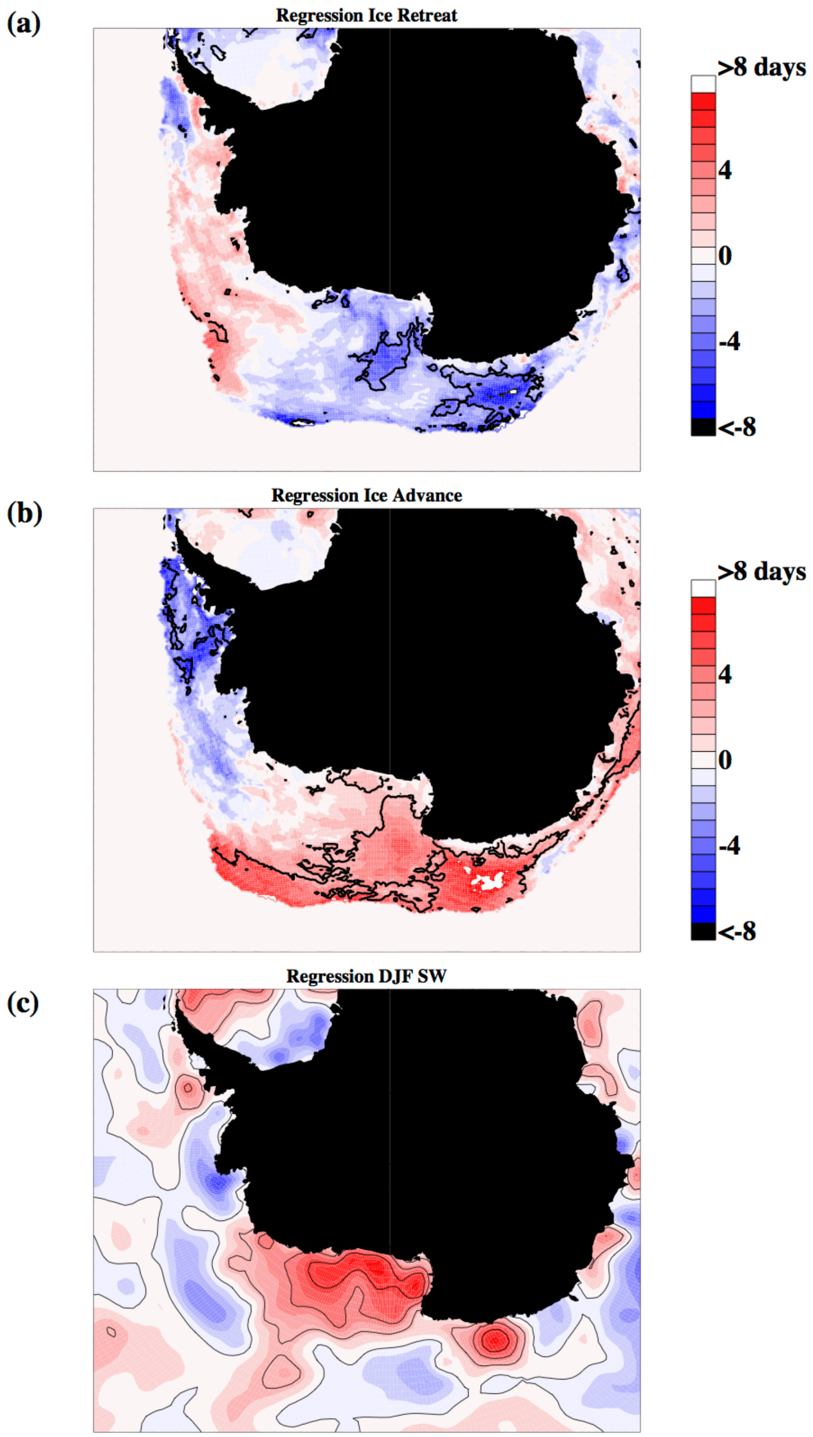

Supplementary Fig. 7. Relationship of environmental properties with October zonal winds. The regression of gridded (a) timing of ice retreat, (b) timing of ice advance, and (c) summer (DJF) net shortwave radiation on normalized October zonal wind anomalies that are averaged over the high trend region shown by the black box on Fig. 5b. On panels (a) and (b), the black contour indicates the 95% significance level. On panel (c), the lined contour interval is 2 W m^{-2} per standard deviation of the wind anomalies.

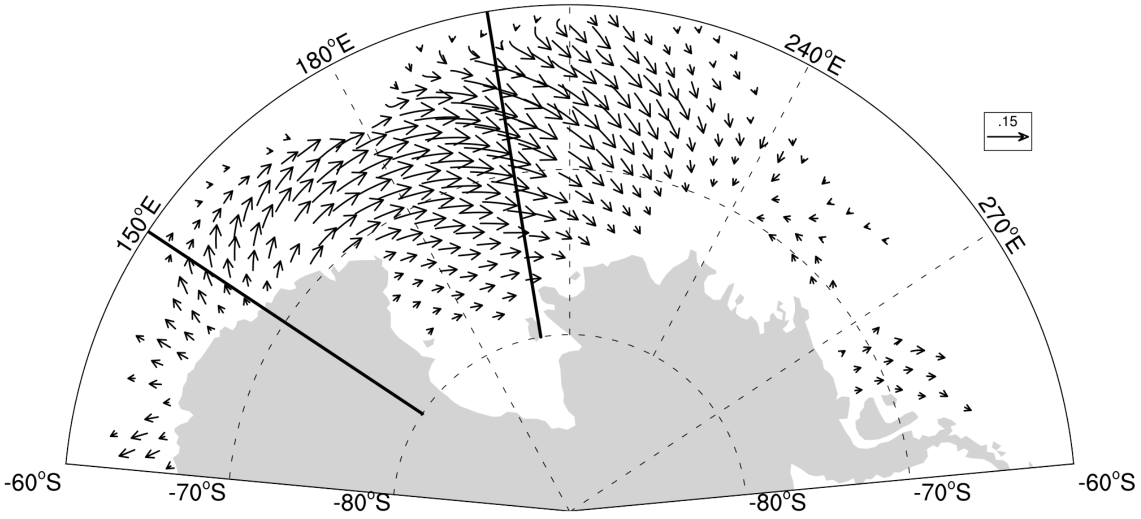

Supplementary Fig. 8. Relationship of ice motion and October zonal winds. The regression of October ice motion on the October zonal wind anomalies averaged over the high trend region shown by the black box on Fig. 5b. Vectors with a magnitude of less than 0.02 are not shown.

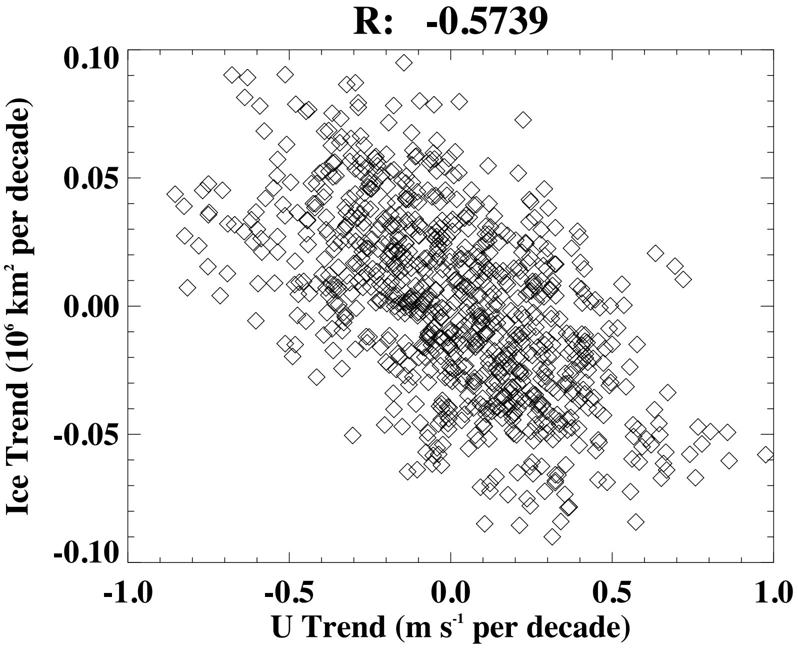

Supplementary Figure 9. Relationship between modeled trends in zonal winds and ice area. The trend in October zonal wind averaged over the black box on Fig. 5b versus the trend in western Ross Sea ice area for all 37-year segments of a 900 year pre-industrial control simulation of the CESM-LE model. The correlation between the two variables, as shown in the plot title, is R=-0.57.

Model Name	Modeling Center	Years	Correlations (% < OBS)	Wind Trends (% < OBS)
ACCESS1-0	Commonwealth Scientific and Industrial Research Org. & Bureau of Meteorology, Australia (CSIRO-BOM)	500	0	4
ACCESS1-3	CSIRO-BOM	500	0	1
Bcc-csm1-1	Beijing Climate Center, China Meteorological Admin (BCC)	500	0	0
CanESM2	Canadian Centre for Climate Modelling and Analysis(CCCma)	1095	0	4
CMCC-CESM	Centro Euro-Mediterraneo per Cambiamenti Climatici (CMCC)	277	0	4
CMCC-CM	CMCC	270	0	9
CMCC-CMS	CMCC	500	0	1
CNRM-CM5	Centre National de Recherches Météorologiques/Centre Européen de Recherche et Formation Avancée en Calcul Scientifique (CNRM-CERFACS)	850	0	3
GFDL-CM3	NOAA Geophysical Fluid Dynamics Lab (NOAA GFDL)	500	0	5
GFDL-ESM2G	NOAA GFDL	500	1	7
GFDL-ESM2M	NOAA GFDL	500	1	4
HadGEM2-CC	Met Office Hadley Centre	240	0	1
IPSL-CM5A-LR	Institut Pierre-Simon Laplace (IPSL)	1000	0	6
IPSL-CM5A-MR	IPSL	300	0	9
IPSL-CM5B-LR	IPSL	300	0	3
MIROC-ESM	Japan Agency for Marine - Earth Science and Technology, Atmos. & Ocean Research Institute (The University of Tokyo), and National Institute for Environmental Studies	630	0	6
MPI-ESM-MR	Max-Planck-Institut für Meteorologie	1000	0	3
MRI-CGCM3	Meteorological Research Institute	500	0	20
NorESM1-M	Norwegian Climate Centre	501	16	6
CESM-LE	Community Earth System Model	900	8	6

Supplementary Table 1. CMIP5 Models. Information on CMIP5 models used in the analysis. The fourth column lists the percent of correlations within a model that are less than or equal to the observed. The fifth column lists the percent of wind trends in the models that are less than or equal to the observed. Analysis is performed on all possible 37-year segments of the model simulations for a direct comparison with the 37-year (1979-2015) observed timeseries.
